# Delays in diagnosis and treatment of pulmonary tuberculosis in patients seeking care at a regional referral hospital, Uganda: a cross sectional study

**DOI:** 10.1186/s13104-019-4616-2

**Published:** 2019-09-18

**Authors:** Winters Muttamba, Samuel Kyobe, Alimah Komuhangi, James Lakony, Esther Buregyeya, Eldad Mabumba, Robert K. Basaza

**Affiliations:** 1 Makerere University Lung Institute, Makerere University College of Health Sciences, Kampala, Uganda; 2 Department of Medical Microbiology, Makerere University College of Health Sciences, Kampala, Uganda; 3 Institute of Public Health and Management, International Health Sciences University, Kampala, Uganda; 4 School of Biotechnology and Laboratory Sciences, Makerere University, Kampala, Uganda; 5 School of Public Health, Makerere University College of Health Sciences, Kampala, Uganda; 6 National Tuberculosis and Leprosy Program, Ministry of Health, Kampala, Uganda; 7 School of Public Health, St. Augustine International University, Kampala, Uganda

**Keywords:** Individual delay, Health facility delay, Pulmonary tuberculosis, Hospital set up, Quality of care, Uganda

## Abstract

**Objective:**

A cross-sectional survey involving 134 pulmonary TB patients started on TB treatment at the TB Treatment Unit of the regional referral hospital was conducted to ascertain the prevalence of individual and health facility delays and associated factors. Prolonged health facility delay was taken as delay of more than 1 week and prolonged patient delay as delay of more than 3 weeks. A logistic regression model was done using STATA version 12 to determine the delays.

**Results:**

There was a median total delay of 13 weeks and 110 (82.1%) of the respondents had delay of more than 4 weeks. Patient delay was the most frequent and greatest contributor of total delay and exceeded 3 weeks in 95 (71.6%) respondents. At multivariate analysis, factors that influenced delay included poor patient knowledge on TB (adjOR 6.904, 95% CI 1.648–28.921; p = 0.04) and being unemployed (adjOR 3.947, 95% CI 1.382–11.274; p = 0.010) while being female was found protective of delay; adjOR 0.231, 95% CI 0.08–0.67; p = 0.007). Patient delay was the most significant, frequent and greatest contributor to total delay, and factors associated with delay included being unemployed, low knowledge on TB while being female was found protective of delay.

## Introduction

Tuberculosis (TB) causes devastating effects among millions of people every year. In 2017, 10 million people developed TB disease and an estimated 1.3 million deaths among HIV negative TB patients and 300,000 deaths among HIV co-infected TB patients were recorded [1].

Uganda is a high HIV/TB prevalent country and just like most sub-Saharan countries, TB control has been complicated by the high HIV prevalence [2]. Delay in presentation, together with delay in making a diagnosis and initiation of treatment is responsible for the increased morbidity and mortality from TB. This is coupled with increased transmission rates in the community [3]. Prompt diagnosis and early initiation of treatment remain key strategies in TB prevention and control. It is thus important that the various types of delays and their underlying factors are well studied if the World Health Organization (WHO) post 2015 strategy and targets are to be realized. This study set out to ascertain the prevalence of individual and health facility delays and associated factors among pulmonary TB patients at Mbale Regional Referral Hospital in Eastern Uganda.

## Main text

### Methods

A cross sectional survey involving all consenting adult patients with pulmonary TB, and started on TB treatment at Mbale Regional Referral Hospital TB treatment unit Uganda in the period September 2015 to February 2016. Importantly, this facility had benefited from country wide roll out of GeneXpert technology that started in 2011.

Clients that had started treatment in the 3 months preceding or during the study were recruited from the TB Unit as they initiated treatment or came back for their drug refill. The study excluded clients that were too ill to respond. A structured interviewer-administered questionnaire was used, and this was adapted from a previous study on TB diagnosis delay in Uganda [4]. Patient delay was defined as the time from the onset of a TB cardinal symptom (cough lasting more than 2 weeks, persistent fevers, noticeable weight loss, excessive night sweats) to the first visit to a health care provider (HCP). Prolonged patient delay was defined as a period of more than 3 weeks. Health facility delay was defined as the time taken from first visit to a HCP up to time of TB diagnosis. Prolonged health facility delay was defined as a delay of more than 1 week. Total delay was taken as the sum of patient delay and health facility delay. Treatment delay was taken as the duration from when the time the diagnosis was made to when the patient was initiated on treatment. A family was used to mean people living under the same roof.

Data gathered were entered into Epi-data software version 2.0.8.56 and exported to Stata software version 12 for analysis. Bivariate analysis using Fishers test was performed on the variables and total delays. Fishers exact test was done given the small numbers under the categories. To ensure more power, we collapsed some categories (occupation, education, action taken at consultation and second health facility consulted) to be able to perform a multivariate analysis of the variables found significant using fishers test.

The study was approved by the Institutional Ethics Review Boards at International Health Sciences University and Mbale Regional Referral Hospital (IRB approval number Number REIRC IN-COM 125/2015).

## Results

Social demographic characteristics of the respondents are presented in Table 1. A total of 134 adults were enrolled into the study. Of these, 61 (45.5%) were male and median age of respondents was 28 years. The median family size was 4 with a range of 1–19.

**Table 1:** Socio-demographic and access characteristics of respondents

Characteristic	No. patients (%)
Age, mean	32
Sex	
Male	61 (45.5)
Female	73 (54.5)
Highest education level attained	
University or higher	8 (6.0)
Primary/middle/secondary	115 (85.8)
Illiterate	11 (8.2)
Occupation	
Technical/professional	20 (14.9)
Peasant	69 (51.5)
Student	29 (21.6)
Unemployed	16 (12.0)
Marital status	
Married	40 (29.9)
Single	52 (38.8)
Divorced/separated	34 (25.4)
Widowed	8 (5.9)
History of smoking	
Never	98 (73.1)
Current smoker	1 (0.8)
Quit smoking	35 (26.1)
Number of household members	
1	10 (7.6)
> 1	124 (92.4)
Residence	
Urban	58 (43.3)
Peri-urban	46 (34.3)
Rural	30 (22.4)
Homeless/displaced	0 (0)

The distribution of delays, individual and health facility factors associated with delay analyzed using Fishers test and logistical regression are presented in Table 2. Median total delay was 13 weeks, with a large total delay of more than 4 weeks in 110 (82.1%) respondents. Patient delay exceeded 3 weeks in 96 (71.6%) respondents, and median patient delay was 11 weeks. Health facility delay exceeded 1 week in 59 (44.0%) respondents, with median being 1 week. Treatment delay was witnessed in 74 (58.7%) respondents.

**Table 2:** Distribution of delays and predictors of delay

Total delay	n (%)	Median	
Delay (> 4 weeks)	110 (82.1)	13 weeks	
No delay (< 4 weeks)	24 (17.9)		
Health facility delay			
Delay (> 1 week)	59 (44.0)	1 week	
No delay (< 1 week)	75 (56.0)		
Patient delay			
Delay (> 3 weeks)	96 (71.6)	11 weeks	
No delay (< 3 weeks)	38 (28.4)		
Treatment delay			
Delay (> 0 days)	74 (58.7)	1 day	
No delay (0 days)	52 (41.3)		

Factors associated with prolonged delay			
Variable	Total delay		p-values (Fishers test)
	Yes	No	
Sex			0.025**
Female	65 (89.0)	8 (11.0)	
Male	45 (73.8)	16 (26.2)	
Occupation			0.001**
Technical/professional	20 (100)	0 (0)	
Peasant	58 (84.1)	11 (15.9)	
Student	17 (58.6)	12 (41.4)	
Unemployed (or HW)	15 (93.8)	1 (6.2)	
Marital status			0.022**
Married	37 (92.5)	3 (7.5)	
Single	36 (69.2)	16 (30.8)	
Divorced/separated	30 (88.2)	4 (11.8)	
Widowed	7 (87.5)	1 (12.5)	
Household size			0.361
Education level			0.699
University or higher	6 (75)	2 (25)	
Primary/middle/secondary	94 (81.7)	21 (18.3)	
Illiterate/read and write	10 (90.9)	1 (9.1)	
Residence			0.336
Urban	48 (82.8)	10 (17.2)	
Peri-urban	40 (87.0)	6 (13.0)	
Rural	22 (73.3)	8 (28.7)	
Smoking			0.835
Never	81(82.7)	17 (17.3)	
Current smoker	1 (100)	0 (0)	
Quit smoking	28 (80.0)	7 (20)	
Stigma			0.019
Health facility whom you sought consultation: second			0.007**
TB centre	22 (64.7)	12 (35.3)	
Public hospital/outpatient clinic	66 (89.2)	8 (10.8)	
Private hospital/clinic	19 (90.5)	2 (9.5)	
PHC well equipped			0.007**
Best	53 (73.6)	19 (26.4)	
Worst	57 (91.9)	5 (8.1)	
Action taken			0.046**
Sputum examination	52 (89.7)	6 (10.3)	
Xray	14 (82.4)	3 (17.6)	
Both sputum and Xray	39 (73.6)	14 (26.4)	
Referral	0 (0)	1 (100)	
Others	5 (100)	0 (0)	

Multivariate analysis		
Variable	Adjusted OR (95% CI)	p > |Z|
Gender		
Female	0.231 (0.080–0.670)	0.007**
Occupation		
Unemployed	3.947 (1.382–11.274)	0.010**
Marital status		
Unmarried	3.364 (0.871–13.001)	0.079
Second health facility consulted		
Private	0.632 (0.177–2.254)	0.479
Knowledge on TB		
No knowledge	6.904 (1.648–28.921)	0.04**
Action taken		
Xray and sputum	2.412 (0.904–6.433)	0.904

Unemployed (student, unemployed/housewife), employed (technical/professional, peasant), unmarried (single, divorced, separated, widower)

** Statistically significant at 0.05 level

At bivariate analysis, factors associated with delay were male gender with being female being protective (p = 0.025), being a professional/technical worker (p = 0.001), being married (p = 0.022), low knowledge on TB (p = 0.001), having a second consultation from a public facility (p = 0.007), poorly equipped health facility as judged by patient (p = 0.007) and having only sputum examination requested (p = 0.046). At multivariate analysis, factors that influenced delay included poor patient knowledge on TB (p = 0.04) and being unemployed (p = 0.010). The median health facility delay was one (1) week. Health facility delay was associated with action taken at consultation, and when both Xray in addition to sputum was done, the number with a delay reduced to 73.5% (39/53); i.e. a reduction of 16.1%.

At multivariate analysis, factors that influenced delay included poor patient knowledge on TB (adjOR 6.904, 95% CI 1.648–28.921; p = 0.04) and being unemployed (adjOR 3.947, 95% CI 1.382–11.274; p = 0.010) while being female was found protective of delay; adjOR 0.231, 95% CI 0.08–0.67; p = 0.007).

## Discussion

This study done at a regional referral hospital determined the magnitude of delay and also the individual and health facility factors associated with the delay. A delay of more than 4 weeks in 82.1% of the patients was noticed with patient delay being the biggest contributor of the total delay. A similar study done in Uganda found health facility delay as the biggest contributor to the delays [3, 4]. This difference could be due to improvements in the diagnostic capacities of many laboratories especially with the introduction of more sensitive tests including the GeneXpert technology could have shortened the health facility delays. Similar studies in different settings have found patient delay as the commonest type of delay [5–7].

Female gender was associated with lower chances of having total delay, different from findings in a study done in Mukono [8] and other studies [9–12]. The reasons for this could be that the females normally have better health seeking behaviors than their male counterparts. Men tend to neglect symptoms until the disease reaches a serious stage, by which time they tend to go directly to public health services without first visiting private health practitioners [13, 14].

Being unemployed in this study was associated with higher odds of having prolonged delay. This could be explained by the financial challenges the unemployed people go through to transport themselves to the diagnostic health facilities and later on pay for their care.

Lack of knowledge was a big predictor of prolonged delay. Our findings are similar to those in the study done in Kampala, Mukono and Wakiso [3, 4] where education was found to be a predictor of delay. Lack of information on TB has been found to be associated with delay in studies done elsewhere in East Africa [15].

As in previous studies [10, 16], there was a strong relation between having a second consultation and total delay similar to our results after bivariate analysis, which was not the case at multivariate analysis. Prior attendance to a clinic or second consultation was also found to be a predictor of delay in a study done in Ethiopia [17].

Over half, 79 (58.7%) of the clients were started on TB treatment a day or more after diagnosis. The policy recommended by the World Health Organization is that treatment be initiated on the same day that the diagnosis is made [18]. Doing this would help cut the cycle of infection and also ensure the initial default rates are reduced as some patients might not return to start treatment.

## Limitation

Recall bias during the interviews was a limitation. We allowed enough time for careful probing and used available records for missing information. Also referral patterns of the patients were not ascertained i.e. how many of the patients were referred by the lower health facilities and how many were self-referred.

## Data Availability

The dataset generated during and/or analysed during the current study are available from the corresponding author on reasonable request.

## Abbreviations

TB: tuberculosis
HIV: human immunodeficiency virus
WHO: World Health Organization
HCP: health care provider
IRB: Institutional Review Board
